# Complete Genome Sequence of Halophilic Yeast *Meyerozyma caribbica* MG20W Isolated from Rhizosphere Soil

**DOI:** 10.1128/genomeA.00127-15

**Published:** 2015-03-19

**Authors:** Jong-Shik Kim, Jeong-Hoon Baek, Nyun-Ho Park, Changhoon Kim

**Affiliations:** a Gyeongbuk Institute for Marine Bioindustry, Uljin, Republic of Korea; b Macrogen, Inc., Seoul, Republic of Korea

## Abstract

Meyerozyma caribbica MG20W was originally isolated from rhizosphere soil on reclaimed land in the Republic of Korea. We describe herein the 10.64-Mbp-long genome sequence of M. caribbica MG20W, which exhibits high salt resistance.

## GENOME ANNOUNCEMENT

Meyerozyma caribbica MG20W is a yeast strain isolated from plant rhizosphere soil of Saemangeum reclaimed land on the west coast of the Republic of Korea. Analysis using a Genome Sequencer FLX system (Titanium; 454 Life Sciences, Branford, CT, USA) resulted in 651,521 high-quality reads assembled using Newbler2.5.3 software (1) into 52 contigs of >500 bp. Paired-end sequencing produced 93,485 reads (mean read length, 2,758 bp).

The genome sequence of M. caribbica MG20W was determined at Macrogen, Inc. (Seoul, Korea) with 454 technology, and was paired read and gapped using Phred 0.990310. The genome size is 10.64 Mbps, and 9 contigs are represented. A total of 227,896,009 bp of raw data was sequenced, representing approximately 20-fold coverage of the M. guilliermondii ATCC 6260 genome. The average G+C content and quinone (high-performance liquid chromatography) were 45.14% and Q-9, respectively.

Gene prediction analysis using the software program Glimmer (2) identified 7,472 open reading frames (ORFs) in the genome, 226 ORFs of tRNA, 9 ORFs of 18S rRNA, 113 ORFs of 8S rRNA, 49 ORFs of lipase, 512 ORFs of transporter, and 8 ORFs of pump.

Comparative genomic analysis revealed that the genome of M. caribbica MG20W is most closely related to that of M. guilliermondii ATCC 6260 (3, 4), sharing 45% of homologous proteins. We found the following gene ontology terms after mapping: biological process, 2,040; cellular component, 1,969; molecular function, 1,207; no hits, 2,256.

### Nucleotide sequence accession numbers.

The sequence data reported in this paper were deposited in the DNA Data Bank of Japan/EMBL/GenBank nucleotide sequence databases with the accession numbers BADS01000001 to BADS01000009.
